# Macrobenthic Biomass Relations in the Faroe-Shetland Channel: An Arctic-Atlantic Boundary Environment

**DOI:** 10.1371/journal.pone.0018602

**Published:** 2011-04-22

**Authors:** Bhavani E. Narayanaswamy, Brian J. Bett

**Affiliations:** 1 Scottish Association for Marine Science, Scottish Marine Institute, Oban, Argyll, United Kingdom; 2 Ocean Biogeochemistry and Ecosystems, National Oceanography Centre, Southampton, United Kingdom; National Institute of Water & Atmospheric Research, New Zealand

## Abstract

The Faroe-Shetland Channel, located in the NE Atlantic, ranges in depth from 0–1700 m and is an unusual deep-sea environment because of its complex and dynamic hydrographic regime, as well as having numerous different seafloor habitats. Macrofaunal samples have been collected on a 0.5 mm mesh sieve from over 300 stations in a wide area survey and on nested 0.5 and 0.25 mm mesh sieves along a specific depth transect. Contrary to general expectation, macrofauanl biomass in the Channel did not decline with increasing depth. When examined at phylum level, two main biomass patterns with depth were apparent: (a) polychaetes showed little change in biomass on the upper slope then increased markedly below 500 m to a depth of 1100 m before declining; and (b) other phyla showed enhanced biomass between 300–500 m. The polychaete response may be linked with a seafloor environment change to relatively quiescent hydrodynamic conditions and an increasing sediment mud content that occurs at c. 500 m. In contrast, the mid-slope enhancement of other phyla biomass may reflect the hydrodynamically active interface between the warm and cold water masses present in the Channel at c. 300–500 m. Again contrary to expectation, mean macrofaunal body size did not decline with depth, and the relative contribution of smaller (>0.25 mm<0.5 mm) to total (>0.25 mm) macrobenthos did not increase with depth. Overall our total biomass and average individual biomass estimates appear to be greater than those predicted from global analyses. It is clear that global models of benthic biomass distribution may mask significant variations at the local and regional scale.

## Introduction

Fauna living in the deep sea depend on organic matter originating from the surface waters to survive. The exceptions to this are the chemosynthetic environments such as cold seeps and hydrothermal vents [1]. Organic matter quality and quantity typically decrease in an exponential fashion as depth and distance from shore increases [2]–[4]. The rate of deposition of organic matter can influence multiple benthic community attributes, including: body size [5], faunal composition [6], trophodynamics [7], community structure and organization [8] to name but a few. In their global study of deep-sea benthic standing stock, Rex et al. [9] found that all faunal groups (excluding bacteria) decreased significantly with increasing depth and distance from shore. They noted that there was very little overlap between the depth - abundance relationships of each faunal group, whereas with biomass, there was considerable overlap among the groups. There were also shifts in dominance by the faunal groups, with the macrofauna dominating upper bathyal regions and the meiofauna dominating lower bathyal and abyssal areas.

Deep-sea animals are thought to have adapted to the decline in input of organic matter in one of two ways. Some communities, namely the macro- and meio-faunal sized organisms show a move towards decreasing body size i.e. miniaturization, with increasing water depth as proposed by Thiel [5], [10]. Other studies have also provided support for this hypothesis e.g. Gage [11] of the macrofaunal community in the Rockall Trough, Soeatert & Heip [12] of the nematode community in the Mediterranean, Soltwedel et al. [13] of the meiofauna in the north-eastern Atlantic, and Kaariainen & Bett [14] of the macro- and meio-benthos of the Northern European Seas. However, the reverse has also been reported with body size increasing (gigantism) in some taxa, such as in isolated crustaceans, e.g. some scavenging amphipods [15], [16] and Isopoda [17], as well as gastropods [18] and nematodes in the Puerto Rico Trench and Hatteras abyssal plain [19].

This work relates to the Census of Marine Life's “Synthesis of Fresh Biomass” project where the main aims have been to i) create a biomass database for the main faunal size groups, ii) to map the global distribution of their biomass, and iii) to compare the relative biomass of different taxa and groups at specific locations or within an ecosystem. Wei at el. [20] highlighted from their global biomass analysis the classic log-linear decrease in biomass with increasing depth, but that they also reported a difference in the rate of decline dependent on the faunal size even though food limitation was the same for each group.

### Background to study area

**Figure 1:**
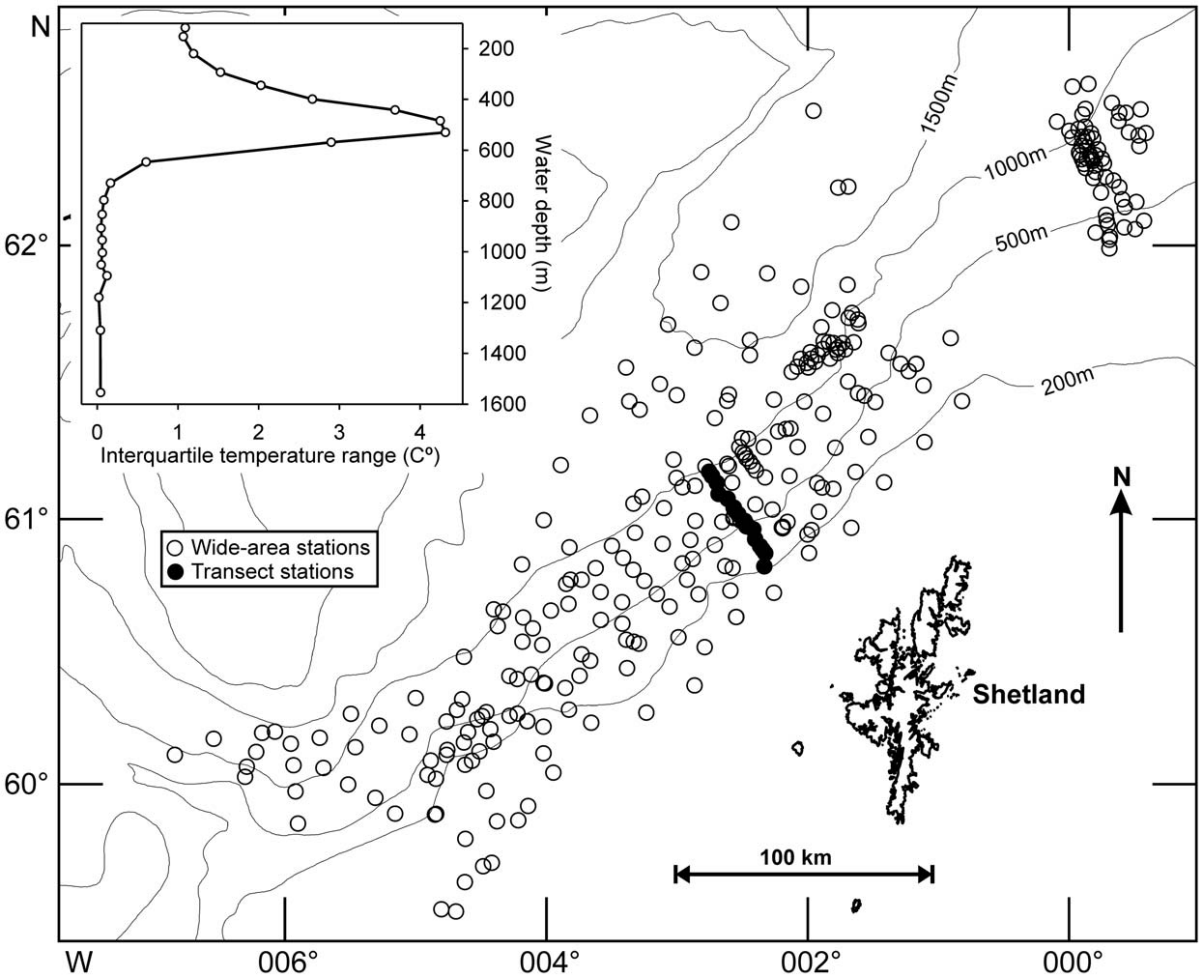
Chart showing the locations of Faroe-Shetland Channel sampling sites. Open circles represent the 329 stations sampled in the wider area survey, filled circles represent the 15 stations of the depth transect. Inset: Bottom water temperature range in the study area (see text for details).

Macrofaunal samples were collected during three cruises over a four year time span (1996–2000) [21]–[23]. A total of 344 stations were sampled for macrofauna in the Faroe-Shetland Channel, ranging in depth from approximately 133 to 1700 m (Figure 1). Many different seabed types and features can be found in the Channel ranging from the intrusion of the North Sea Fan, a contourite band, iceberg ploughmarks, dense gravel cover and mud diapirs [23]–[25]. The Channel also experiences a wide temperature range (see Figure 1 inset) as a result of a number of water masses converging in this area [26]–[28]. Cold, dense Norwegian Sea water flows towards the south-west underlying warmer North Atlantic water flowing in a north-easterly direction [28]. Not only does the Channel experience fluctuating water temperature, but the speed and degree at which the water temperature can change is remarkable; in some instances a 7 C° change in temperature in one hour [29]. Internal waves propagating along the channel at the warm-cold water boundary are thought to cause of the rapid change in temperature [30] and can displace water masses vertically by as much as 100 m [31]. Hosegood et al. [32] also revealed the presence of solibores propagating up the slope in the Channel, which in turn leads to substantial sediment resuspension.

## Methods

### Field sampling and data

We include two sets of samples in this analysis (Figure 1): (i) wide-area survey, 329 stations sampled, primarily in a stratified random sampling design (by water depth and geographic area; [29]) that assessed >0.5 mm macrobenthos over three cruises (1996, 1998, 2000 [21]–[23]), and (ii) 15 detailed bathymetric transect stations, sampled in both 1996 and 1998, that considered both >0.5 and >0.25 mm macrobenthos.

Macrofaunal samples were collected using a variety of sampling gear; an hydraulically damped Megacorer [33] (171 wide-area and 7 transect stations), a modified USNEL box corer [34] (85 wide-area and 4 transect stations) and a Day grab [35] (73 wide-area and 4 transect stations) were used in this preferential order as the sample quality varied between these devices [29], [36]. However, seafloor type dictated which equipment was used. At the shallower stations, the Day grab was used as the risk of damaging the box corer and Megacorer on the cobbles and boulders that are abundant on the seafloor was extremely high. Macrofaunal samples were collected from the entire contents of the Day grab (∼5 l equivalent to 0.1 m^2^), a 0.1 m^2^ insert placed inside the box core or eight pooled cores from the Megacorer (0.063 m^2^). The samples were sieved on 0.5 mm mesh sieves for the wide-area survey and on 0.5 mm and 0.25 mm mesh stacked sieves for the depth transect samples. In all cases, the residue was preserved in 10% borax buffered formalin. Rose Bengal was added to the preserved samples to aid with the sorting process.

Samples were also collected for the analysis of sedimentological parameters, e.g. mean grain size, mud content (particles <63 µm) and total organic carbon (for detailed methodology see [29]). We have also compiled bottom-water temperature data for the general study area from the online archive of the British Oceanographic Data Centre (www.bodc.ac.uk; 99 CTD casts taken 1996–2001). These data were pooled and various statistics derived for water depth horizons corresponding to those used for the macrobenthos analyses (see below).

### Abundance and biomass determinations

Depending on the sieve size fraction, two techniques were employed to sort the macrofauna. The >0.5 mm fraction was sub-sampled and this sub-sample distributed on a white tray and sorted using an illuminated bench magnifier. The original sample continued to be sub-sampled until it had been completely sorted. The >0.25 mm<0.5 mm fraction was sorted using a flotation technique (usually employed for the extraction of meiofauna [37]). The original sample was re-washed and small fractions were added to a Ludox™ solution (colloidal silica). The resulting mixture was gently stirred and left to settle for approximately 20 minutes. The surface layer of the Ludox solution, containing the macrofauna, was gently poured through a 0.25 mm sieve leaving behind as much of the residue as possible. The Ludox was collected and added to the residue; the process was repeated until no further macrofauna appeared in three consecutive extractions. A subset (20 of 30) of the remaining residues was checked, these confirmed 100% extraction. The recovered macrofauna were then sorted using a WILD M5 binocular microscope. In both size fractions, the fauna were sorted and counted into five major groups, annelids, crustaceans, echinoderms, mollusks and others. Blotted wet weight biomass was measured using a Sartorius BP221S balance. Tube-dwelling specimens were removed from their tubes prior to weighing, although in the case of the very small, fragile fauna, this was not always practical or possible.

### Statistical analyses

As the environment dictated the use of three different sampling gears, a sampler performance related statistical analysis was undertaken first [38]. We employed analysis of variance (ANOVA; [39]) to make an initial assessment of overall variation in faunal density and biomass estimates between gears. As macrofaunal standing stocks might vary systematically with water depth (e.g. [9]) and any gear bias be proportionate to standing stock, we also undertook an analysis of covariance (ANCOVA; [39]), i.e. the influence of gear type on log transformed macrofaunal abundance and biomass was tested using water depth as a covariate. We subsequently used the ANCOVA method to calculate sampler bias correction factors (see e.g. [36]) based on the resultant adjusted mean values.

To summarise the wide-area survey results we have analysed our data in water-depth horizons, such that each horizon contains 15 samples (deepest horizon, n = 14). This partition of the data essentially follows the original depth-based stratified random sampling design adopted by Bett [29]. For presentation we have calculated geometric mean and 95% confidence intervals [40] based on log(x+1) transformed data (e.g. density, biomass, mean individual biomass). For comparability we similarly present the results of the detailed transect study as geometric mean values of the 1996 and 1998 data.

Spearman's rank correlation (see e.g. [41]) was employed to investigate potential relationships between the biomass of the different phyla and faunal size groups and a range of environmental variables.

## Results

### Sampler bias

**Figure 2:**
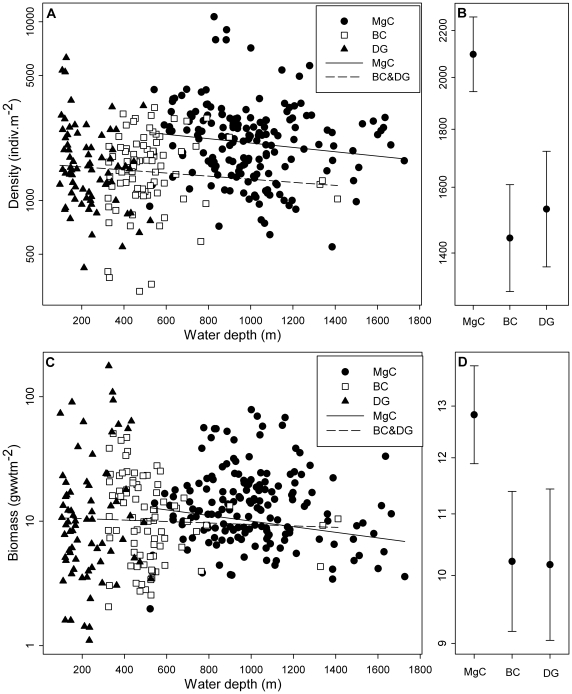
Variation in macrofaunal standing stocks (>0.5 mm, uncorrected) with depth in the Faroe-Shetland Channel. Complete wide-area survey data is shown, keyed to sampler type (MgC, Megacorer, n = 171; BC, box corer, n = 85; DG, Day grab, n = 73). (A) and (C) sample data with separate regression lines for MgC and combined BC&DG data. (B) and (D) Geometric mean and 95% confidence intervals of standing stock by sampler type irrespective of water depth.

The complete macrofaunal standing stock (density and biomass) dataset for the wide area survey (329 stations) is illustrated in Figure 2, with values for each gear type shown separately. Analysis of variance was carried out to examine the potential influence of gear type on standing stock (log transformed) irrespective of water depth, both density (F 19.72, p≪0.001) and biomass (F 3.24, p<0.05) varied significantly with gear type. Figures 2B and 2D illustrate geometric mean and 95% confidence intervals for standing stock by gear type. In both cases the Megacorer result is substantially higher than both the Day grab and the box corer. There is very little variation between the Day grab and the box corer results. This is in accord with the earlier gear comparisons carried out by Bett [38], who found no significant differences (p>0.05) between macrofaunal standings stock estimates from Day grab and box corer samples collected in the 300–400 m water depth range. Similarly, Bett [38] did record statistically significant differences (p<0.05) between macrofaunal standings stock estimates from box core and Megacorer samples collected in the 500–800 m water depth range.

Bett's [38] use of depth-restricted comparisons between gear types was an acknowledgement of potential systematic variation in macrofaunal standing stocks with water depth. Analysis of covariance (ANCOVA) provides a means to extend this comparison over the full water depth range of the wide-area survey. To proceed with this analysis we first amalgamated the Day grab and box corer data into a single gear type (DG&BC), we believe this is reasonable, since neither we nor Bett [38] detected any significant differences in standing stock estimates from these two gears. Standing stock estimates from DG & BC and Megacorer (MgC) samples all exhibit a slight negative trend with depth (see Figures 2A and 2C). For faunal density the regression coefficients (DG & BC, −8.64×10^−5^; MgC, −1.22×10^−4^) are not significantly different between gear types (t 0.3419, p>0.05). Similarly for biomass, the regression coefficients (DG & BC, −5.86×10^−5^; MgC, −1.17×10^−4^) are not significantly different between gear types (t 0.3426, p>0.05). Given the common slopes of these relationships we carried out the ANCOVA. This indicated that gear type (DG&BC or MgC) had a statistically significant influence on measured macrofaunal standing stock (density: F 30.04, p≪0.001; biomass: F 5.91, p<0.05). Depth-adjusted mean values derived from the ANCOVA suggest gear correction factors as follows:
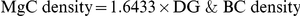


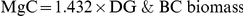
An ANCOVA was undertaken on the gear adjusted values, with gear type being found to have no significant effect (density: F 0.00, p = 1; biomass F 0.00 p = 0.968). On the basis of these depth-adjusted mean values, Day grab and box core samples only recovered 60% of the abundance and 70% of the biomass collected in Megacorer samples. The ANCOVA also indicated that while density varied significantly with depth (F 4.38, p<0.037) biomass did not (F 1.23, p>0.05).

### General variations in biomass

**Figure 3:**
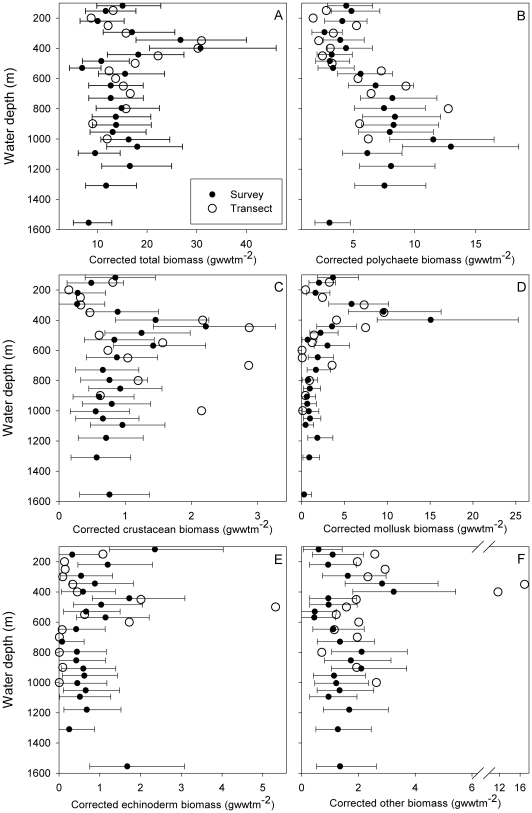
Variation in corrected biomass (>0.5 mm) with depth. (A) Total biomass. (B) Polychaete biomass. (C) Crustacean biomass. (D) Mollusk biomass. (E) Echinoderm biomass. (F) Other taxa biomass. Solid circles indicate the wide-area survey depth bands (geometric mean and 95% confidence interval), open circles indicate the depth transect stations (geometric mean).

**Figure 4:**
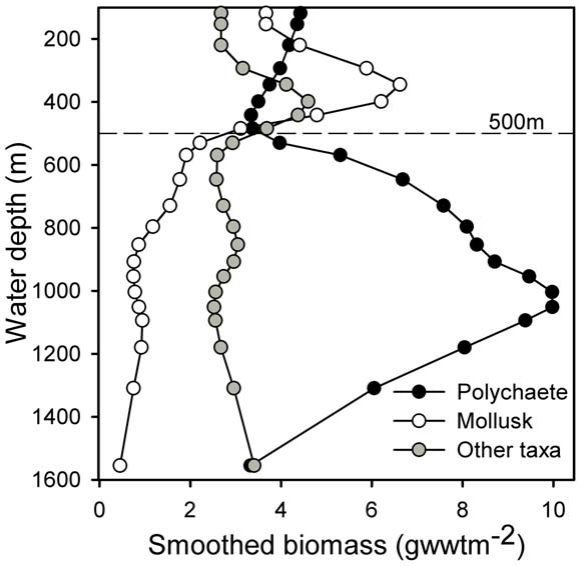
Summary of depth-related patterns in macrofaunal biomass distribution with depth. Wide-area survey geometric mean biomass (>0.5 mm, corrected) of selected major taxa, shown after depth-ordered smoothing (4253H, twice method; see e.g. Velleman [53]).

Variations in macrofaunal biomass (>0.5 mm) with depth show broadly comparable trends whether examined as measured biomass (supporting Figure S1) or as gear bias corrected biomass (Figure 3). Data from the detailed transect generally follow the trends of the wide-area survey, athough exhibit a greater range of variation (see e.g. Figures 3E and 3F) as is to be expected given their different sample sizes (transect n = 2, wide-area n = 15). There appear to be two main patterns in the depth distributions of biomass. For total, crustacean, mollusk, echinoderm and combined other taxa biomass (Figures 3A, 3C–F) there is no marked monotonic relationship with depth but all, to a greater or lesser degree, exhibit some increase in biomass in the 300–500 m depth range. In contrast, polychaete biomass remains relatively constant to 500 m, thereafter increasing to 1000 m before declining to the full depth of the survey. These patterns are most readily seen in smoothed data from the wide-area survey (Figure 4).

**Figure 5:**
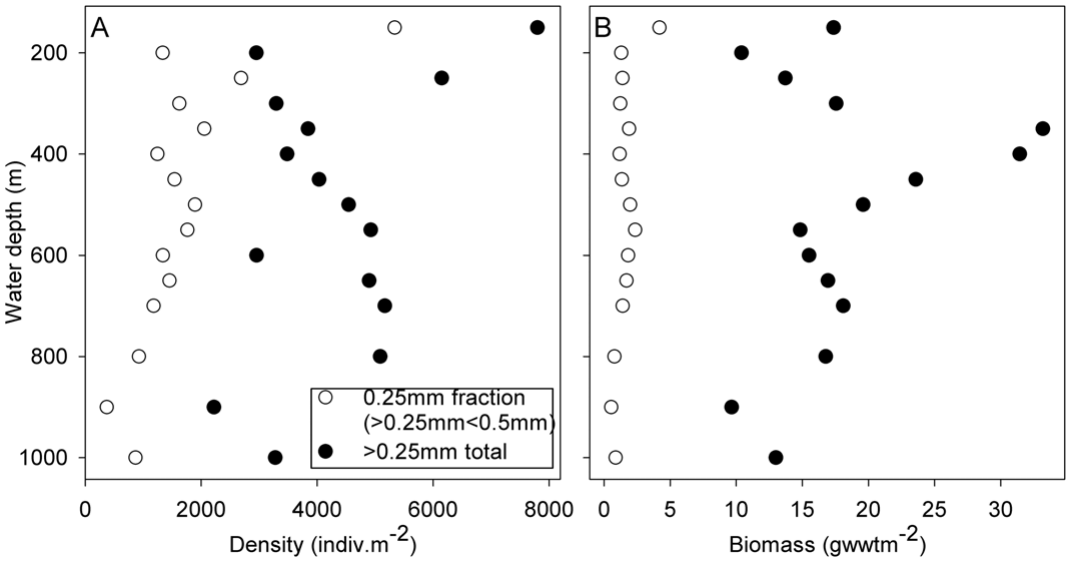
Corrected density and biomass values of the >0.25 mm<0.5 mm and >0.25 mm fractions for the transect stations. (A) Density. (B) Biomass.

Variations in macrofaunal density and biomass (>0.25 mm, transect stations) with depth are illustrated with the contribution of the 0.25 mm fraction alone in Figure 5. As expected, the 0.25 mm fraction can comprise a substantial fraction of total density (>0.25 mm), ranging from 17–68% of total density with a mean of 38% on a per station basis. The contribution to total biomass (>0.25 mm) is substantially less, ranging from 4–24% with a mean of 10%. Consequently, the biomass (>0.25 mm) depth trend (Figure 5B) is very similar to that of the >0.5 mm biomass (Figure 4A), with a local increase in the 350–400 m depth range. The proportional contribution of the 0.25 mm fraction to total (>0.25 mm) standing stock declines with increasing depth; however the decline is not statistically significant in the case of biomass, but is highly significant in the case of density (Spearman's rank p<0.001). These relationships vary among the major taxa (not shown), for example, the proportional contribution of the 0.25 mm fraction polycheates declines significantly in the case of both density (p<0.001) and biomass (p<0.002), the same is true for crustacean density (p<0.001) and biomass (p<0.05). However, the proportional contribution of the 0.25 mm fraction mollusk increases significantly in the case of biomass (p<0.05).

### Biomass and environment relationships

Potential relationships between macrofaunal biomass and a range of environmental parameters were examined by non-parametric correlation and the results summarized in Table 1. Overall, polychaete biomass exhibited the strongest and most consistent relationships. In the three groups of tests (wide-area >0.5 mm, transect >0.5 mm, and transect >0.25 mm) polychaete biomass was significantly (p<0.05) positively correlated with water depth, and sediment mud content, and negatively correlated with all three bottom water temperature parameters (minimum, maximum and range). In the wide-area survey data an opposing trend is apparent for crustacean biomass: significant negative correlations with depth and mud content, significant positive correlations with all temperature parameters.

**Table 1:** Spearman's rank correlations of macrofaunal biomass (corrected) and environmental variables.

Survey (sieve fraction)	Taxon group	Water depth	Mean sediment grain size	Total organic carbon	Mud content	Minimum water temperature	Maximum water temperature	Water temperature range
Wide-area (>0.5 mm)	Polychaete	**0.532**	**−0.577**	**0.452**	**0.582**	**−0.529**	**−0.532**	**−0.572**
(n = 22)	Crustacean	**−0.727**	**0.680**	−0.398	**−0.722**	**0.543**	**0.727**	**0.700**
	Mollusk	−0.090	0.313	−0.145	−0.142	0.072	0.090	**0.434**
	Echinoderm	−0.239	0.259	−0.051	−0.233	0.335	0.239	0.240
	Other taxa	0.251	−0.350	0.071	0.278	−0.127	−0.251	−0.219
	Total	−0.184	0.025	−0.076	−0.098	0.174	0.184	0.240
Transect (>0.5 mm)	Polychaete	**0.757**	−0.500	0.465	**0.775**	**−0.738**	**−0.756**	**−0.627**
(n = 15)	Crustacean	0.504	−0.418	0.481	**0.596**	**−0.522**	−0.493	−0.068
	Mollusk	**−0.521**	0.286	−0.109	−0.371	0.466	0.520	**0.670**
	Echinoderm	−0.471	**0.596**	−0.206	−0.368	0.416	0.463	**0.692**
	Other taxa	−0.457	0.246	−0.403	**−0.568**	0.440	0.468	0.397
	Total	−0.104	−0.150	0.227	0.007	0.120	0.105	**0.638**
Transect (>0.25 mm)	Polychaete	**0.732**	−0.482	0.497	**0.779**	**−0.711**	**−0.729**	**−0.629**
(n = 15)	Crustacean	0.379	−0.239	0.401	0.493	−0.422	−0.365	−0.182
	Mollusk	−0.439	0.218	0.079	−0.236	0.418	0.436	**0.643**
	Echinoderm	−0.443	**0.593**	−0.202	−0.336	0.395	0.434	**0.635**
	Other taxa	−0.096	−0.086	−0.052	−0.207	0.063	0.105	0.245
	Total	0.293	−0.518	**0.521**	0.439	−0.266	−0.288	0.281

Of the environmental parameters tested, bottom water temperature produced almost twice as many significant correlations as any other correlate. Significant (p<0.05) positive relationships were recorded for crustacean, mollusk and echinoderm biomass, and negative relationships with polychaete biomass. This is in broad accord with the two main patterns in the depth distributions of biomass noted above. For total, crustacean, mollusk, echinoderm and combined other taxa biomass (Figures 3A, 3C–F), a local increase in biomass in the 300–500 m depth range corresponds with the region of increased bottom water temperature variation (see Figure 1 inset). In contrast, polychaete biomass remains low to 500 m before increasing in the region of minimal bottom water temperature variation and relatively constant low temperatures (c. −1°C).

## Discussion

### Biomass-depth relationship

**Figure 6:**
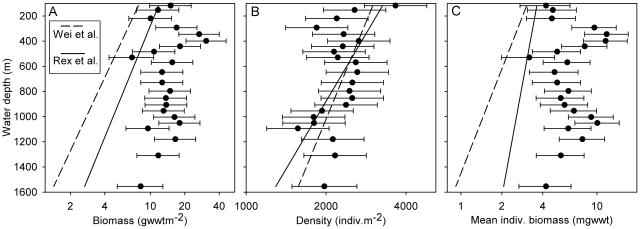
Variation in corrected macrofaunal biomass, density and mean individual biomass (>0.5 mm) with depth. (A) Biomass. (B) Density. (C) Mean individual biomass. Geometric mean and 95% confidence interval are shown for each parameter (wide-area survey data). Vertical lines are the global regressions of these parameters on depth established by Rex et al. [9] and Wei et al. [20].

Our observations of macrobenthic biomass relations in the Faroe-Shetland Channel (FSC) do not generally conform to the expectation of a logarithmic decline in benthic standing stocks with increasing water depth and / or distance from shore [42], [9]. Figure 6 plots our estimates of standing stocks with predictions from the log-linear regressions of global standing stocks on water depth established by Rex et al. [9] and Wei et al. [20]. In the case of macrobenthic population density, our observations lie close to those of the global regressions (Figure 6B). However, our estimates of biomass (Figure 6A) and average individual biomass (Figure 6C) appear to be systematically higher than the global predictions. The global regressions encompass many sources of variation, both methodological (e.g. sampler type, sieve mesh size, biomass determination technique) and environmental (e.g. overhead surface primary production, proximity to sources of laterally advected organic matter). Consequently, significant local / regional variations in the biomass-depth relationship may be masked in global compilations. In the present case we might suggest that the elevated biomass in the FSC represents the above global average surface primary production in this region (see e.g. [43]). However, FSC macrofaunal density is broadly consistent with the global prediction, i.e. not suggestive of above global average surface primary production. This apparent contradiction leads also to the above prediction values for mean individual biomass in the FSC. Kaariainen & Bett [14] made a detailed study of benthic body size distributions in the deep FSC (1600 m), concluding that the fauna was characterised by small individuals, as expected in the deep sea, and that mean individual biomass was a poor, oftern misleading descriptor of underlying body size distributions.

### Biomass-environment relationships

Two primary patterns in biomass distribution with depth and potential links to environmental factors were evident in our data. Firstly, for total, crustacean, mollusk, echinoderm and combined other taxa biomass (Figures 3A, 3C–F) there is enhanced biomass in the 300–500 m depth range corresponding with the region of increased bottom water temperature variation (see Figure 1 inset). This environmental heterogeneity may well directly enhance macrobenthic diversity (see [25]) but is unlikely to influence biomass in the same manner. However, temperature range may serve as a proxy for the intensity of the local hydrodynamic regime. Hosegood and van Haren [32] and Hosegood, Bonnin and van Haren [44] report the occurrence of solibores in the Faroe-Shetland Channel, graphically illustrating the extreme temperature fluctuations associated with the passage of these internal wave-like features (see e.g. Fig 9A of Hosegood and van Haren [32]). These solibores are also highly significant in local sediment resuspension and transport [44], consequently, they may enhance / focus organic matter supply in the 300–500 m depth band and improve conditions for filter-feeding macrobenthos.

**Figure 7:**
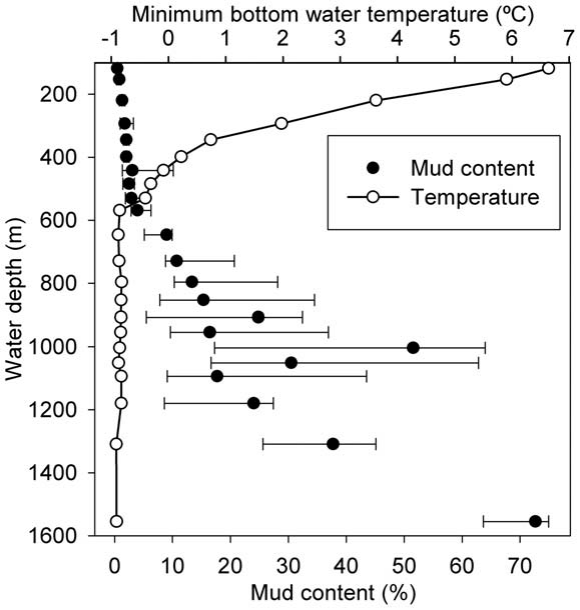
Variation in sediment mud content (<63 µm) and minimum bottom water temperature with depth. Median and interquartile range are shown for mud content.

The second of the primary patterns in biomass distribution with depth is that exhibited by the polychaetes (Figure 3B), that is consistenly low from c. 100–550 m, where bottom waters are comparatively warm or highly variable in temperature. Polychaete biomass then begins to increase substantially where there is the transition to the region experiencing sub-zero temperature waters (c. 600 m; Figure 7). Polychaete biomass peaks at c. 1050 m, the transition from slope to Channel floor, before declining to the maximum water depth surveyed (c. 1700 m). The sedimentary environment also changes markedly at c. 600 m water depth, with a distinct and rapid increase in sediment mud content from median values <4% at shallower depths, to a median >70% at the maximum depth of our survey (Figure 7). This change likely reflects the transition from the dynamic conditions of the upper slope, where substantial sediment resuspension and transport may occur (e.g. [44]), to more quiescent conditions that permit an increasing accumulation of fine-grained sediments. The increasing mud content may allow more extensive development of the infaunal deposit-feeding macrobenthos, typified by the polychaetes. Polychaete biomass, both in the wide-area survey and the transect study, did show a significant positive correlation with sediment mud content (Table 1).

### How important is the small size fraction?

The choice of sieve mesh size for the study of deep-sea macrobenthos has been a somewhat contentious issue [33]. The influence of mesh size (e.g. 0.5 mm versus 0.25 mm) on faunal parameters is thought to be highly variable [45], having limited impact on biomass, modest effect on diversity, but a major influence on abundance. In our study the contribution of the 0.25 mm fraction alone to the >0.25 mm total ranged from 4–24% in the case of biomass and 17–68% for abundance among the transect stations. Noteably, the contribution of the 0.25 mm fraction to total >0.25 mm abundance declines significantly with water depth (Spearman's rank correlation, −0.853, p<0.001). This is contrary to the general expectation of the increasing importance of smaller body size classes with depth (see e.g. [5]). Similarly, our estimates of mean individual biomass (Figure 6C) do not exhibit any consistent decline with water depth. Note, however, that mean individual biomass may be a poor and potentially misleading description of the underlying population body size distribution [14]. The latter authors contrasted a shallow-water (150 m) North Sea site with a deep-water (1600 m) Faroe-Shetland Channel site, recording near identical values for metazoan (macro- and meio-benthos) mean individual biomass despite a very substantial and significant shift in body size distributions to smaller classes at the deep-water site.

### Sampler bias

Undersampling by box cores relative to multiple corers has been long-established in the case of meiobenthos studies (see e.g. [36]) but is also evident in macrobenthos work. Bett & Gage [46] detailed statistically significant sampler bias between Megacorer and box corer macrobenthos samples from the Faroe-Shetland Channel region and in the adjacent Rockall Trough (see also [33]). The data presented by Hughes and Gage [47] from other Rockall Trough sites also show statistically significant sampler bias between Megacorer and box corer macrobenthos samples. The effect is also evident among box core and multiple core macrobenthos samples examined by Blake and Narayanaswamy [48] collected in the Southern Ocean during the ANDEEP project [49]. In an ‘ideal world’, a single sampler capable of collecting high quality quantitative macrobenthos samples would be employed. However, for a range of practical reasons this may not always be possible. For example, the seafloor environment in the Faroe-Shetland Channel region is extremely heterogeneous, ranging from soft deep-sea muds, through sand bodies, and areas of complete gravel cover, to a cobble and boulder strewn ‘iceberg ploughmark zone’ on the upper slope and a shelf-edge environment with a minimal coarse sediment veneer overlying consolidated boulder clay [50]–[52]. No single current benthic sampling device is capable of routine practical operation in this range of seafloor habitats.

### Conclusions

Our macrofaunal biomass estimates for the Faroe-Shetland Channel are somewhat higher than those that have been predicted at a global level. They indicate how important it is to be aware of siginificant biomass variations at both local and regional scales when considering global models. They also suggest the need for a greater understanding of how methodology (e.g. sampler bias) and environmental factors specific to any region may cause considerable variation. In addition our results highlight varying patterns among the biomass-depth relationships of individual phyla. In our study, polychaetes exhibited a markedly different response to all other taxa, seemingly related to a switch in hydrodynamic regime and corresponding change in the sedimentary environment. Our results concerning macrofaunal body size also depart from general expectation, mean individual body size did not decline with depth, and the relative contribution of smaller (>0.25 mm<0.5 mm) to total (>0.25 mm) macrobenthos did not increase with depth. These measures may, however, be a poor description of the underlying body size structure, which may be best assessed with full size spectra studies.

## Supporting Information

Figure S1 **Variation in measured (uncorrected) biomass with depth.** (A) Total biomass. (B) Polychaete biomass. (C) Crustacean biomass. (D) Molluskan biomass. (E) Echinoderm biomass. (F) Other taxa biomass. Solid circles indicate the wide area survey depth bands (geometric mean and 95% confidence interval), open circles indicate the depth transect stations (geometric mean).
(TIF)
